# Noninvasive phase mapping of persistent atrial fibrillation in humans: Comparison with invasive catheter mapping

**DOI:** 10.1111/anec.12527

**Published:** 2017-12-22

**Authors:** Andreas Metzner, Erik Wissner, Alexey Tsyganov, Vitaly Kalinin, Michael Schlüter, Christine Lemes, Shibu Mathew, Tilmann Maurer, Christian‐Hendrik Heeger, Bruno Reissmann, Feifan Ouyang, Amiran Revishvili, Karl‐Heinz Kuck

**Affiliations:** ^1^ Asklepios Klinik St. Georg Hamburg Germany; ^2^ Petrovsky National Research Centre of Surgery Moscow Russia; ^3^ EP Solutions SA Yverdon‐les‐Bains Switzerland; ^4^ Vishnevsky Institute of Surgery Moscow Russia

**Keywords:** atrial fibrillation, noninvasive mapping, rotors, phase mapping

## Abstract

**Background:**

A novel noninvasive epicardial and endocardial electrophysiology system (NEEES) to identify electrical rotors and focal activity in patients with atrial fibrillation (AF) was recently introduced. Comparison of NEEES data with results from invasive mapping is lacking.

**Methods:**

Six male patients (59 ± 11 years) with persistent AF underwent cardiac mapping with the NEEES, which included the creation of isopotential and phase maps. Then patients underwent catheter mapping using a PentaRay NAV catheter and the CARTO 3 system. Signals acquired by the catheter were analyzed by customized software that applied the same phase mapping algorithm as for the NEEES data.

**Results:**

In all patients, noninvasive phase mapping revealed short‐lived electrical rotors occurring 1.8 ± 0.3 times per second and demonstrating 1–4 (mean 1.2 ± 0.6) rotation cycles. Most of these rotors (72.7%) aggregated in 2–3 anatomical clusters. In two patients, focal excitation from pulmonary veins was observed. Invasive catheter mapping in the dominant rotor aggregation sites and in the three control sites demonstrated the presence of electrical rotors with properties similar to noninvasively detected rotors. Spearman's correlation coefficient between rotor occurrence rate by noninvasive and invasive mapping was 0.97 (*p *<* *.0001). Mean rotors' cycle length at dominant aggregation sites, scores of their full rotations, and the proportion of rotors with clockwise rotation were not significantly different between the mapping modalities.

**Conclusion:**

In patients with persistent AF, phase processing of unipolar electrograms recorded by catheter mapping could reproduce electrical rotors as characterized by NEEES‐based phase mapping.

## 1 INTRODUCTION

Atrial fibrillation (AF) is the most common cardiac arrhythmia with an increasing impact on global health (Chugh et al., 2014). In 1998 Haissaguerre et al. demonstrated that ectopic focal impulses originating mainly from the pulmonary veins (PV) are a major cause of AF initiation (Haïssaguerre et al., 1998). Based on this finding, ablation procedures focusing on PV isolation have been developed and are currently accepted as the cornerstone of all AF ablation strategies (Calkins et al., 2012). However, the success rates of catheter ablation in patients with persistent AF are significantly lower than that of paroxysmal AF ablation (Calkins et al., 2012). One of the reasons is that the mechanisms underlying persistent forms of AF are unclear.

Despite decades of intensive research, the mechanisms of perpetuating AF are still not fully understood. The contradictory “multiple wavelets” and “leading circle” mechanistic theories of AF were proposed in various studies (Jalife, Berenfeld, Skanes, & Mandapati, 1998).

The development of optical mapping of action potentials in vitro resulted in new insights into the underlying mechanism of myocardial fibrillation. Optical mapping studies in animal models and explanted human hearts demonstrated that the spatiotemporal organization of atrial and ventricular fibrillation may be associated with periodically observed spiral waves (electrical rotors) (Gray, Pertsov, & Jalife, 1998; Hansen et al., 2015; Mandapati, Skanes, Chen, Berenfeld, & Jalife, 2000).

In those studies, a special technique of optical action potential processing and imaging of rotors, namely, phase mapping, was used. The phase mapping principle is based on utilizing the phase component of action potential signals instead of the signal itself (Clayton & Nash, 2015). The phase of an action potential is supposed to be related to the electrical activation‐recovery state of the myocardium at the point of signal registration. On phase maps, the pivot point of a rotor is located at the convergence of the “iso‐phase” lines where the phase value is undetermined. In terms of phase mapping, the pivot point of a rotor may be called the phase singularity (PS). The calculation of PS locations by special algorithms is used to determine and track rotor “cores” (Clayton & Nash, 2015).

The crucial point in phase map construction is the computational method for the phase of cardiac electrical signals. The most common method is based on the Hilbert transform (Clayton & Nash, 2015).

Since optical phase mapping is challenging in humans, methods for transformation of optical action potentials into phase values were adopted for unipolar electrograms (EGs) (Umapathy et al., 2010). This approach was successfully applied for mapping of ventricular fibrillation in human hearts during surgical procedures (Nash et al., 2006). Recently, Narayan, Krummen, Clopton, Shivkumar, & Miller (2013) reported an innovative phase mapping technology based on basket catheters to identify and locate AF drivers, including electrical rotors and focal impulses. Catheter ablation of persistent AF patients guided by this technology resulted in restoration and maintenance of sinus rhythm recovery in most patients (Narayan et al., 2013).

In parallel, noninvasive electrocardiographic imaging (ECGI) based on the inverse problem of electrocardiography was introduced; initial results of noninvasive mapping for AF patients were reported by Cuculich et al. (2010). Based on body‐surface ECG mapping, the ECGI procedure enables reconstruction of unipolar EGs of thousands of points on the myocardium (Revishvili et al., 2015; Rudic et al., 2016).

It should be noted that phase mapping based on unipolar EGs requires complex signal processing (Clayton & Nash, 2015). Several research groups have used different methods, starting from band‐pass filtering to more advanced sinusoidal recomposition (Kuklik et al., 2015). A recent study demonstrated that various signal processing methods may significantly affect the results of phase mapping obtained by body surface ECG mapping and direct contact mapping using the local unipolar EGs (Rodrigo et al., 2014). Thus, both the accuracy of noninvasive phase mapping in persistent‐AF patients and its compatibility with invasive phase mapping is urgently needed.

The objective of the present study was to directly compare, in patients with persistent AF, the same phase processing algorithm at identical filtering frequencies applied to invasive multipolar catheter mapping and noninvasive cardiac mapping.

## 2 METHODS

### 2.1 Patients

Six consecutive symptomatic patients with drug‐refractory persistent AF were consented to noninvasive electrophysiology mapping and invasive catheter mapping, with subsequent ablation. Demographic data are listed in Table 1. Preprocedural diagnostics included a 12‐lead ECG and transesophageal echocardiography. All patients gave their written informed consent and all patient information was anonymized.

**Table 1:** Baseline characteristics of atrial fibrillation (AF) patients enrolled in the study

Patient #	Age	Gender	Diagnosis	Comorbidities	AF duration	AAD	LA, mm	LV EF, %	CHADS_2_‐VASc Score	Catheter procedure
1	50	M	Persistent AF	HT, Stroke (TIA)	6 months	β‐blockers	46	45	4	PVI
2	45	M	Persistent AF		3 months	β‐blockers	44	55	0	PVI
3	70	M	Persistent AF	HT, CAD, Stroke (TIA)	7 months	β‐blockers	45	55	4	PVI
4	55	M	Persistent AF	HT	4 months	β‐blockers	46	50	1	PVI + roof line + CFAE
5	62	M	Persistent AF	CAD	5 months	β‐blockers	56	55	1	PVI
6	74	M	Persistent AF	HT, CAD	1 month	β‐blockers	54	55	3	PVI + roof line

AAD, antiarrhythmic drugs; CAD, coronary artery disease; CFAE, complex fractioned atrial electrograms; LV EF, left ventricular ejection fraction; HT, arterial hypertension; LA, left atrial diameter; PVI, pulmonary veins isolation; TIA, transient ischemic attack.

This study was approved by the Institutional Review Board of Asklepios Klinik St. Georg.

### 2.2 Noninvasive epicardial and endocardial electrophysiology system

The methodology of ECGI with the noninvasive epicardial and endocardial electrophysiology system (NEEES) has been previously described in detail (Chaykovskaya et al., 2015; Tsyganov et al., 2017; Wissner et al., 2016, 2017). In brief, up to 224 MRI‐compatible unipolar ECG electrodes in special arrays were fixed onto the patient's torso, followed by a thoracic contrast MRI (Magnetom Avanto, 1.5T, Siemens, Erlangen, Germany) performed on the same day. Based on the MRI data, the 3D epi‐ and endocardial biatrial geometry was reconstructed applying the NEEES proprietary software (EP Solutions SA, Yverdon‐les‐Bains, Switzerland). In the electrophysiology laboratory, the electrode arrays were connected to the NEEES multichannel ECG amplifier followed by ECG recordings of 10 min in duration applying a bandwidth of 0.05–500 Hz, a sampling rate of 1,000 samples/s. Body‐surface ECG data was processed by the NEEES applying its inverse problem solution software in combination with MRI‐derived anatomical data from the heart and torso. About 2,500 local unipolar EGs were projected onto the epicardial and the endocardial surfaces of the atria. The resulting isopotential and phase maps were reconstructed on the 3D geometry of the heart and displayed as individual frames or activation movies.

In order to represent the spatially distributed reentrant electrical activity of the cardiac tissue, the instantaneous phase values of action potential signals are calculated at each point of the cardiac surface; then the phase values are interpolated and depicted by color coding on the heart surface. A series of instantaneous phase maps can be visualized by animation.

### 2.3 Noninvasive phase mapping

During ECG processing only TQ intervals >350 milliseconds (msec) containing multiple AF waves were selected for phase analysis. QRS‐T segments were not processed. Band‐pass filtering of EGs with a 3–9 Hz Butterworth filter was used before phase calculation.

The following formulas were applied to calculate phase components of the unipolar signals:

$$\varphi \left(t\right)=\mathrm{atan}2[\bar{Hu(t)},\;\overset{¯}{u}\left(t\right)],$$

$$\overset{¯}{u}\left(t\right)=u\left(t\right)-u_{\mathrm{mean}}$$

$$\bar{Hu(t)}=Hu\left(t\right)-u_{\mathrm{mean}}$$

$$u_{\mathrm{mean}}=\left(u_{\max}+u_{\min}\right)/2$$

(*t *= time; φ(*t*) = the signal phase component, *u*(*t*) = unipolar EGs; and *H *= Hilbert transform operator shifting the signal phase by $\frac{\pi }{2}$).

The Hilbert transform was calculated based on a fast Fourier transform (FTT). We performed FTT using the 2.5‐s time windows for both noninvasively reconstructed and invasively recorded electrograms to maintain similarity. The two‐argument atan2 (*x*,* y*) function is a variation of the arctangent function that evaluates the angle between the positive *x*‐axis and the point given by the (*x*,* y*) coordinates. Phase maps were generated based on signal phase distribution on the atrial 3D model surfaces. The line of phase jump from −π to π was considered as a phase front. The PS was calculated using the convolution kernels method (Umapathy et al., 2010). The calculation and plotting of PS were used for identification and tracking of rotor core movement.

### 2.4 Noninvasive identification of AF rotors and focal activity

We identified rotors and focal activity points using isopotential and phase maps while taking into account the specific properties of reconstructed local unipolar EGs and controlling the ECG in standard leads.

A stable rotor was defined as:

1. Phase front rotation of more than 360°;
2. Rotor core meandering not exceeding 25 mm along the atrial surface during one rotation cycle.

Additionally, we checked for the existence of a rotor in areas of interest by its correlation with the rotational pattern in the isopotential map (rotation of isopotential lines around the pivot point), as well as by the occurrence of a distinct F wave in standard ECG‐leads I, II or III.

Atrial fibrillation (AF) focal activity criteria were set as follows:

1. Clear centrifugal pattern of the negative potential spreading in the isopotential maps;
2. QS morphology of local unipolar EGs at focal activity sites.

In order to identify the most frequent locations of rotors we used cumulative maps created according to the principles described above. For each patient, at least 50 consecutive TQ intervals in standard ECG leads were processed. Rotor locations were determined as the centers of PS meandering areas (i.e. the average position of the rotor core during the rotation cycles). Positions of focal impulses were determined as a starting site of centrifugal negative‐potential spreading. Locations of rotor or focal activity were marked on the 3D atrial models.

Segmentation of the atria was performed to study the spatial distribution of AF drivers. The LA was divided into following segments: left atrial appendage (LAA), left superior PV (LSPV) ostium, left inferior PV (LIPV) ostium, anterior, roof, posterior, inferior, right superior PV (RSPV) ostium, right inferior PV (RIPV) ostium, and septum. The right atrium (RA) was divided into following segments: right atrial appendage (RAA), lateral, posterior, cavo‐tricuspid isthmus (CTI), superior vena cava (SVC) ostium, inferior vena cava (IVC) ostium, coronary sinus (CS) ostium, and septum.

### 2.5 Interventional procedure

Invasive mapping was conducted in the electrophysiology laboratory immediately after noninvasive mapping. All procedures were performed under deep sedation using midazolam, fentanyl, and propofol.

The double transseptal puncture was performed under fluoroscopic guidance using a modified Brockenbrough technique and two 8.5F transseptal sheaths (SL1, St. Jude Medical, Inc., St. Paul, MN, USA). All patients underwent invasive 3D reconstruction of the atria using the CARTO 3 system (Biosense Webster, Inc., Diamond Bar, CA, USA) and a 20‐pole mapping catheter (PentaRay NAV 2‐6‐2 D‐curve, Biosense Webster, Inc.). The catheter contains 20 electrodes spaced equidistantly along five branches which spread out from the catheter's deflectable tip. The catheter's contact with the endocardium generates mapping data from a wide anatomical area.

The mapping catheter was positioned in the RA sequentially along the lateral and posterior wall and septum of the RA, in the RAA, SVC and IVC ostia and CTI. The mapping catheter was positioned in the LA sequentially along the roof, anterior, posterior and inferior wall and septum of the LA, in the LAA and PV ostia. Special attention was paid to the segments with frequent rotor presence as identified by noninvasive mapping. Multiple EGs records from the mapping catheter's 20 poles were performed by the CARTO 3 system in each of the atrial segments mentioned.

Pulmonary veins (PV) isolation (PVI) was performed in all patients (*n* = 6). In addition, complex fractionated atrial electrogram (*n* = 1) and linear (*n* = 2) ablation were performed.

### 2.6 Invasive data acquisition and reconstruction of phase maps

Following ablation, CARTO 3 data, including the 3D model in form of a polygonal mesh, coordinates of the mapping catheter poles, bipolar and unipolar EGs was exported. The data were processed applying customized software to create invasive phase maps.

Unipolar and bipolar EGs of the catheter's poles and standard ECG data were exported in fragments of 2.5 s. All TQ ECG intervals >350 msec were selected from these fragments for subsequent processing. As in the NEEES analysis, QRS‐T intervals were not analyzed. Based on the catheter EGs, 3D phase maps were generated on the anatomical atrial models created by the CARTO 3 system. This included interpolation of unipolar EGs from the catheter poles to the mesh nodes of the CARTO 3 anatomical models applying the radial basis function method (Buhmann, 2003). Preliminary tests using personalized atrial geometrical models and noninvasively obtained isopotential maps demonstrated that such interpolation provides for relative errors not exceeding 5%.

The same algorithms and parameter settings including signal filtering and Hilbert transform calculation as described for noninvasive phase mapping were used to generate the invasive phase maps.

Phase maps of atrial segments of interest covered by the mapping catheter (total area ≈ 9.6 cm^2^) were analyzed with respect to the direction of rotor rotation, cycle length, and rotor occurrence rate and subsequently compared with the respective data obtained by the NEEES.

### 2.7 Statistical analysis

Continuous variables are presented as mean ± standard deviation (*SD*); they were compared by Mann‐Whitney's *U* test. Categorical data are presented as numbers and percentages; comparisons were made by chi‐square or Fisher's exact test.

To analyze the spatial distribution of rotors the following values were calculated:

1. The proportion of rotors observed in each atrial segment using the formula:

η_*i*_ **= *m*_*i*_/*n*, where *m*_*i*_ is the number of rotors observed in the given atrial segment during the observation period, *n* is the total number of observed rotors, and *i* is the index of the atrial segment;

1. Rotor occurrence rate in each atrial segment using the formula:

ν_*i*_ **= *m*_*i*_/*T*, where *m*_*i*_ is the number of rotors observed in the given atrial segment during the observation period *T*, and *i* is the index of the atrial segment.

The proportion of rotors revolving in CW or CCW direction, mean cycle length of rotors, and the mean number of complete rotations were also calculated for any given atrial segment. The regional distribution of rotors observed in the NEEES study was characterized by the rotor occurrence rate in each atrial segment.

The Spearman's rank correlation coefficient for rotor occurrence rates was calculated in order to estimate the similarity of the results obtained by noninvasive and invasive mapping in each atrial segment where both mapping methods were implemented.

All tests with *p *<* *.05 were considered statistically significant. Statistical analysis was performed using STATISTICA 10 (Statsoft, Inc., Tulsa, OK, USA).

## 3 RESULTS

### 3.1 NEEES

**Figure 1:**
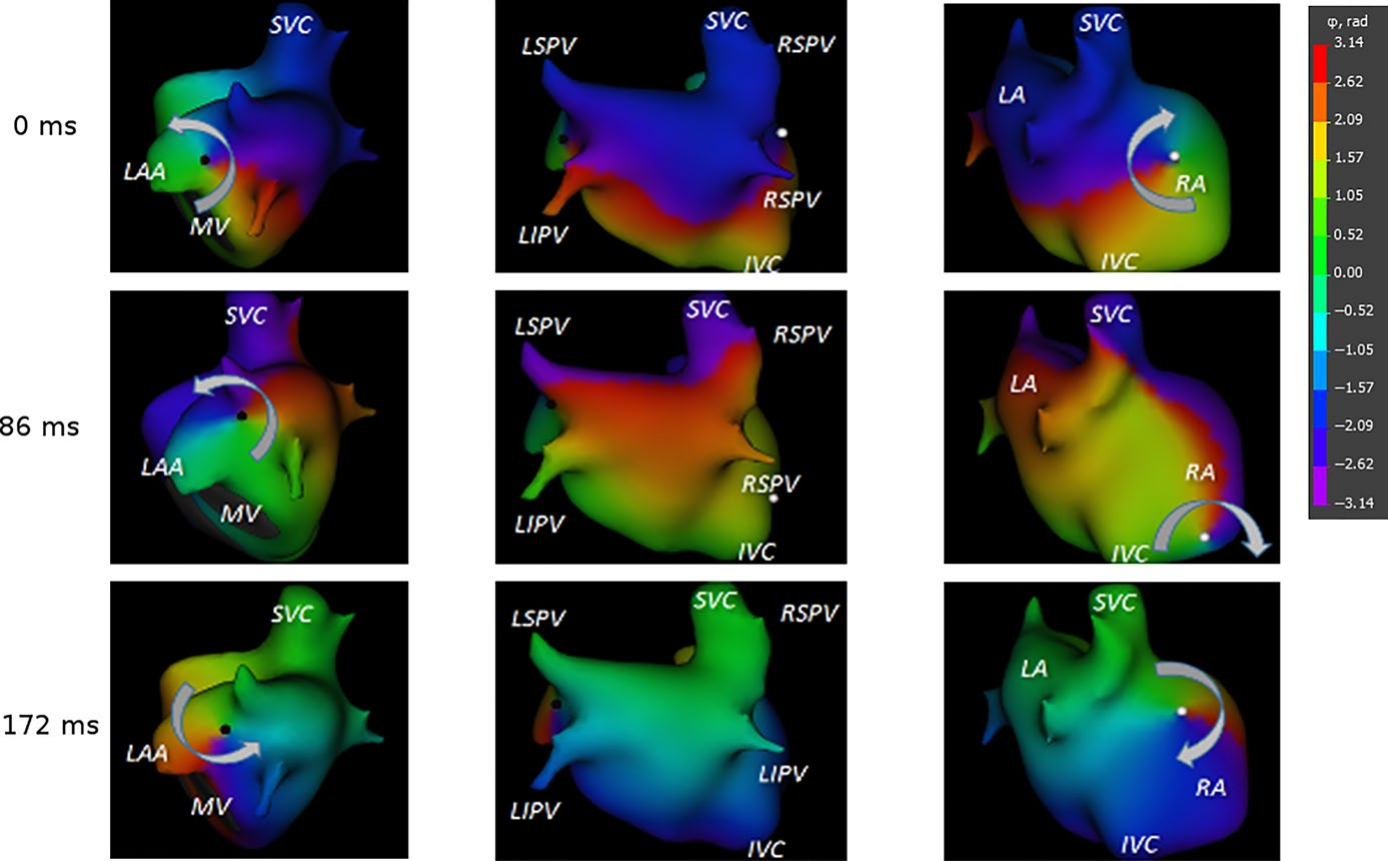
Typical reentrant excitation patterns during atrial fibrillation (AF) visualized by noninvasive phase mapping. Unclosed phase front (the border between red and purple colors) is seen. The phase singularities, marked by white and black points are located at the front ends. The phase front is rotating around phase singularities forming a pair of rotors with opposite chiralities, rotating clockwise and counterclockwise. At the same time, the phase singularities (rotor cores) meander along the atrial surfaces (near the left atrial appendage [LAA] and at the lateral wall of the right atrium [RA])

In all patients, noninvasive phase mapping demonstrated unclosed phase fronts moving over the atrial surfaces. The PS was located at the front ends. The activation pattern could be described as a reentrant movement of the phase front around the PS serving as pivot points. At the same time, PS points also moved along the atrial surface. Different types of PS movements were observed—either rapidly drifting or slowly meandering in a local area. In the latter case, the activation pattern was present as a figure‐of‐eight shaped reentry; the phase front ends organized as a pair of rotors with opposite chiralities, rotating clockwise and counterclockwise (Figure 1). The activation pattern described above was present in approximately 70% of the observation time. In the remainder, a second unclosed phase front with a second pair of PS was present. Interactions and collisions of the phase fronts were observed.

In general, during the total observation period (i.e., the sum of all analyzed TQ intervals) of 123,222 msec in six patients, a single PS‐pair was observed during 87,816 msec (71.3%) and stable rotors with the slow meandering of PS was observed during 35,292 msec (28.6%). The time of single PS‐pair per patient ranged from 63.8% to 72.7% of the total observation time. The observation time of rotor pattern per patient ranged from 21.6% to 33.5% (Table 2). The rotor meandering distance during one full rotation was 12.3 ± 6.2 mm.

**Table 2:** Properties of noninvasively determined rotors

	Patient 1	Patient 2	Patient 3	Patient 4	Patient 5	Patient 6
Observation time^a^, msec	20,790	17,226	19,002	22,285	20,642	23,277
Single pair of PS, msec	14,059 (67.6%)	12, 304 (71.4%)	12,123 (63.8%)	16,204 (72.7%)	14,573 (70.6%)	15,886 (68.3%)
Rotor's stability time, msec	6,374 (30.7%)	5,778 (33.5%)	6,249 (32.9%)	4,823 (21.6%)	5,072 (24,6%)	6,996 (30.1%)
Rotors, *n*	44	36	35	30	32	45
CW rotors, *n* (%)	27 (64.3)	19 (52.8)	12 (34.3)	14 (46.7)	17 (53.1)	16 (35.6)
Full rotor rotations, *n*	1.2 ± 0.4	1.1 ± 0.4	1.2 ± 0.4	1.3 ± 0.6	1.3 ± 0.4	1.2 ± 0.5
1 full rotation, *n* (%)	38 (90.5)	31 (86.1)	28 (80.0)	26 (86.7)	27 (84.4)	34 (75.6)
2 full rotations, *n* (%)	6 (9.5)	5 (13.9)	6 (17.1)	4 (13.3)	5 (15.6)	8 (17.8)
3 full rotations, *n* (%)	0	0	1 (2.9)	0	0	2 (4.4)
4 full rotations, *n* (%)	0	0	0	0	0	1 (2.2)
Rotor cycle length, msec	145 ± 18	161 ± 22	178 ± 24	160 ± 15	159 ± 19	155 ± 11
Meandering distance during 1 cycle, mm	13.8 ± 5.2	15.7 ± 6.1	16.4 ± 5.6	14.7 ± 4.7	15.4 ± 5.3	14.8 ± 4.0
Aggregation sites, *n*	2	2	2	2	2	3
Rotors at aggregation sites, *n* (%)	24 (57.1)@ RSPV roof7 (16.7)@ inferoseptal LA	15 (41.7)@ posterior LA7 (19.5)@ SVC	15 (42.9)@ LSPV14 (40)@ lateral RA	14 (46.7)@ posterior LA10 (33.3)@ RSPV	14 (43.8)@ lateral RA12 (37.5)@ posterior LA	11 (24.4)@ anterior LA9 (20)@ left post. LA8 (17.8)@ right post. LA

PS, phase singularity; CW, clockwise; LA, left atrium; LSPV, left superior pulmonary vein; post., posterior; RA, right atrium; RSPV, right superior pulmonary vein; SVC, superior vena cava.

a Sum of analyzed TQ intervals.

Occasionally we observed some differences in phase pattern between the epicardium and endocardium. At some point in time, the distance between PS on the epicardial and endocardial aspects of the atria exceeded 20 mm. This difference was observed mostly coming together with the rapid drifting of PS. However, when the excitation patterns agreed with rotor definition criteria, the differences in behavior of the phase front and phase singularities on epicardium and endocardium were not significant. In these cases, the distances between the PS arising on both atria aspects ranged from 2 to 6 mm.

During the total observation period of 123,242 msec, a total of 222 rotors were identified in the six patients, with the number of rotors identified per patient ranging between 30 and 45. The mean rotor occurrence rate was 1.81 ± 0.30 per second, with a range of 1.35–2.12 per second (Table 2).

The rotors live cycles varied. The most stable rotor performed 4 rotations. A total of 184 rotors (82.9%) had one full rotation, 34 rotors (15.3%) had two full rotations, 3 rotors had three (1.4%) full rotations, and only 1 rotor had four full rotations. The mean number of full rotations was 1.2 ± 0.6. The proportion of rotors with CW rotation direction was 47.3% (105/222; 95% confidence interval 40.6%–54.1%). The mean rotor cycle length was 160 ± 15 msec.

**Figure 2:**
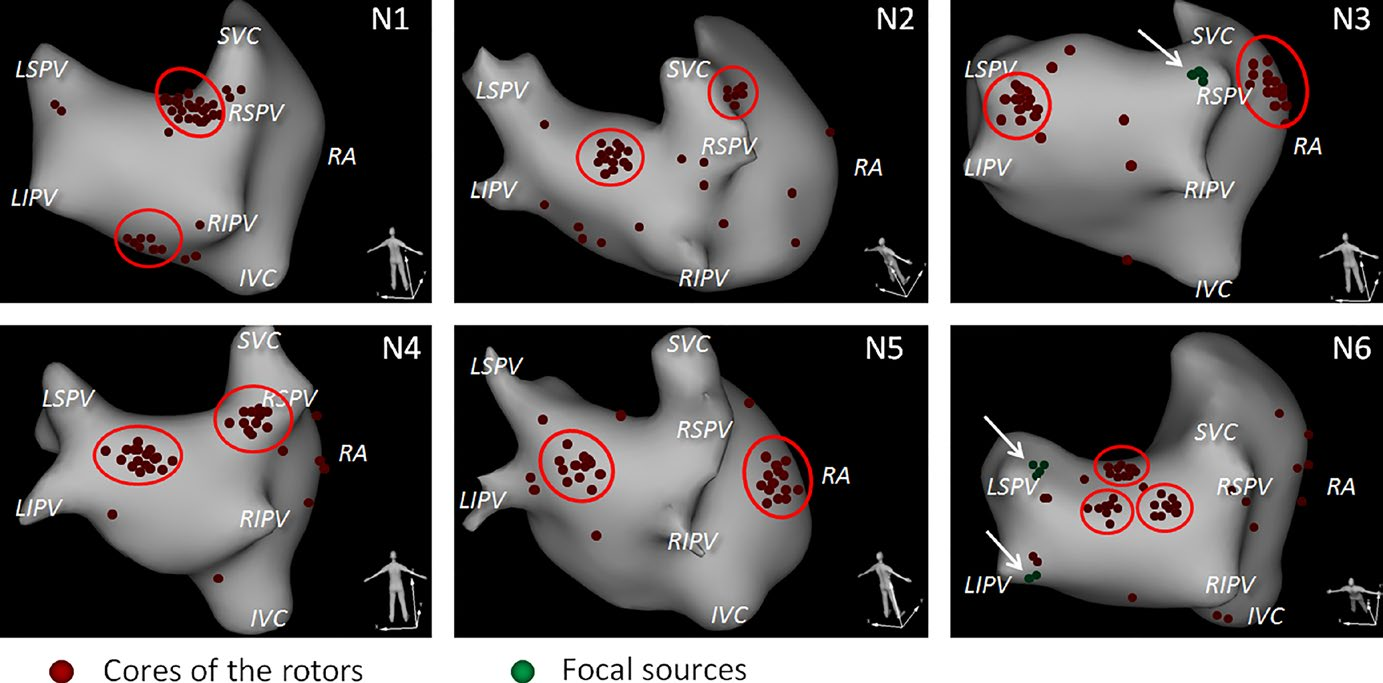
Spatial distribution of rotor cores and focal sources (novel noninvasive epicardial and endocardial electrophysiology system [NEEES]). Most rotors aggregated in few discrete clusters. In five patients two clusters were observed, in one patient three clusters. In two patients (N3 and N6) the focal‐type activation patterns originating from the pulmonary veins were imaged

In spite of the overall stochastic fibrillation pattern, a fairly high organization was observed. The spatial distribution of rotors was not uniform. In general, rotors were observed more frequently in the LA, 164 rotors (73.9%) in the LA versus 58 rotors (26.1%) in the RA. Moreover, in all patients, the areas in which rotors were observed more often (aggregation sites or clusters) than in other areas could be identified (Figure 2). We defined the aggregation site as a zone <10 cm^2^, where >15% of rotors were observed.

In one patient three sites of rotor aggregation were identified, whereas in the other five patients only two rotor aggregation sites were present. Generally, 72.1% (160/222; 95% confidence interval 65.7%–77.9%) of all rotors belong to these sites. Ten rotor aggregation sites were found in the LA: in the posterior region (*n* = 4 patients), in the RSPV region (*n* = 2), in the LSPV region (*n* = 1), in the anterior region (*n* = 1), and in the inferior region (*n* = 1). Three aggregation sites were found in the RA: in the lateral wall of the RA (*n* = 2), and in the SVC region (*n* = 1) (Table 2).

In two patients, in addition to rotors, multiple episodes of centrifugal excitation spreading from the RSPV (*n* = 1), from the LSPV, and from the LIPV (*n* = 1) were observed. These patterns were studied at noninvasively reconstructed isopotential and isochronal maps and appeared to be similar to focal activity episodes.

### 3.2 Invasive catheter mapping

We had to exclude from analysis about 15% of the EGs fragments obtained by the PentaRay catheter because of poor recording quality (poor contact of the catheter electrodes with the endocardium). The total duration of analyzed fragments of EGs recorded by the PentaRay catheter is presented in Table 3.

**Table 3:** Observation times, numbers of rotors, and rotor occurrence rates by novel noninvasive epicardial and endocardial electrophysiology system (NEEES) and catheter mapping in selected atrial segments

		Patient 1		Patient 2		Patient 3		Patient 4		Patient 5		Patient 6	
		NEEES	Catheter	NEEES	Catheter	NEEES	Catheter	NEEES	Catheter	NEEES	Catheter	NEEES	Catheter
LA ant.	Obs. time	20,790	–	17,226	–	19,022	–	22,285	–	20,642	**–**	23,277	9,329
	Rotors, *n*	3	–	0	–	0	–	0	–	1	**–**	11	5
	Occ. rate	0.14	–	0.0	–	0.0	–	0.0	–	0.05	**–**	0.47	0.54
LA roof	Obs. time	20,790	7,931	17,226	6,908	19,022	7,620	22,285	8,470	20,642	7,988	23,277	8,861
	Rotors, *n*	7	2	1	0	0	0	0	0	0	0	2	2
	Occ. rate	0.34	0.25	0.06	0.0	0.0	0.0	0.0	0.0	0.0	0.0	0.09	0.23
LA post.	Obs. time	20,790	8,255	17,226	7,156	19,022	7,437	22,285	8,135	20,642	8,144	23,277	9,074
	Rotors, *n*	1	0	15	6	1	0	14	4	12	4	17	5
	Occ. rate	0.05	0.0	0.87	0.84	0.05	0.0	0.63	0.49	0.58	0.49	0.73	0.55
LA inf.	Obs. time	20,790	8,032	17,226	7,044	19,022	7,688	22,285	8,370	20,642	7,912	23,277	9,125
	Rotors, *n*	9	3	3	1	2	1	0	0	1	0	1	0
	Occ. rate	0.43	0.37	0.17	0.14	0.11	0.13	0.0	0.0	0.05	0.0	0.04	0.0
LSPV	Obs. time	20,790	–	17,226	–	19,022	7,832	22,285	–	20,642	–	23,277	–
	Rotors, *n*	2		1	–	15	5	0	–	1	–	2	–
	Occ. rate	0.10	–	0.06	–	0.79	0.64	0.0	–	0.05	–	0.09	–
RSPV	Obs. time	20,790	8,324	17,226	–	19,022	–	22,285	–	20,642	–	23,277	–
	Rotors, *n*	17	5	3	–	0	–	10	–	0	–	2	–
	Occ. rate	0.82	0.60	0.17	–	0.0	–	0.45	–	0.0	–	0.09	–
RA lat.	Obs. time	20,790	‐	17,226	–	19,022	–	22,285	–	20,642	8,346	23,277	–
	Rotors, *n*	0	–	1	–	14	–	2	–	14	6	5	–
	Occ. rate	0.0	–	0.06	–	0.74	–	0.09	–	0.68	0.72	0.22	–

Observation (Obs.) time in msec; Occurrence (Occ.) rate in 1/sec.

ant., anterior; inf., inferior; LA, left atrium; RA, right atrium; LAA, left atrial appendage; lat., lateral; LSPV, left superior pulmonary vein; post., posterior; RSPV, right superior pulmonary vein.

**Figure 3:**
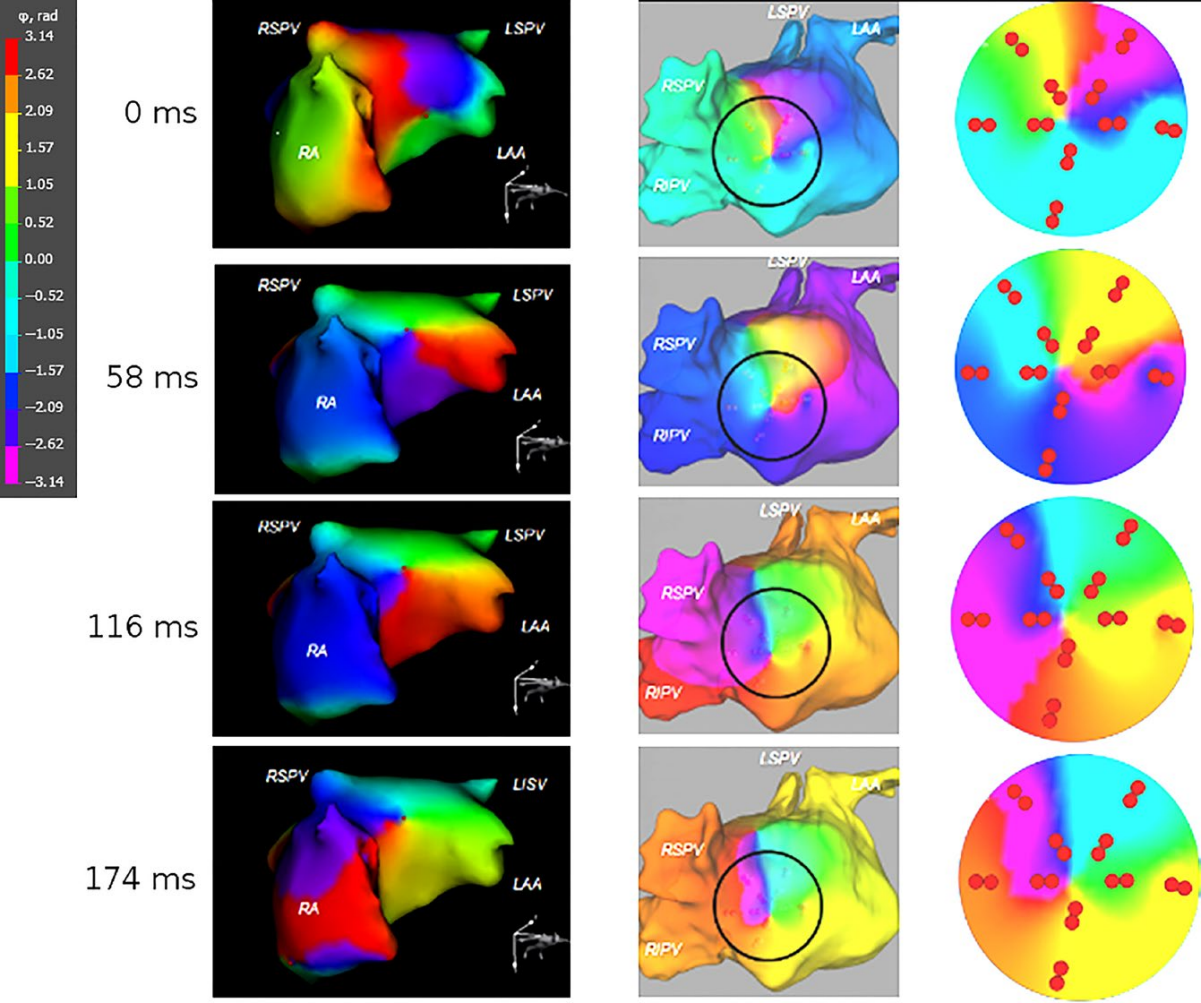
Noninvasively and invasively imaged rotors during atrial fibrillation (AF). The phase maps obtained by novel noninvasive epicardial and endocardial electrophysiology system (NEEES) (left‐hand panels) and catheter mapping (middle and right‐hand panels) demonstrated a rotor in a similar atrial location rotating in the same clockwise direction

In all patients, both local unipolar and bipolar EGs were highly fragmented and did not allow for accurate reconstruction of activation patterns without utilization of phase methods. In contrast, at phase maps, reentrant patterns fulfilling established rotor criteria were observed (Figure 3).

During a total catheter mapping period (i.e. the sum of observation times for each segment in the six patients) of 177,981 msec, a total of 50 rotors were detected. In parallel, 146 rotors were detected by NEEES in the same segments for the same patients; the sum of noninvasive observation times for each segment was 453,347 msec. Thus, in the selected atrial segments the rotor occurrence rates were 0.28 per second by PentaRay mapping and 0.32 per second by noninvasive mapping.

**Figure 4:**
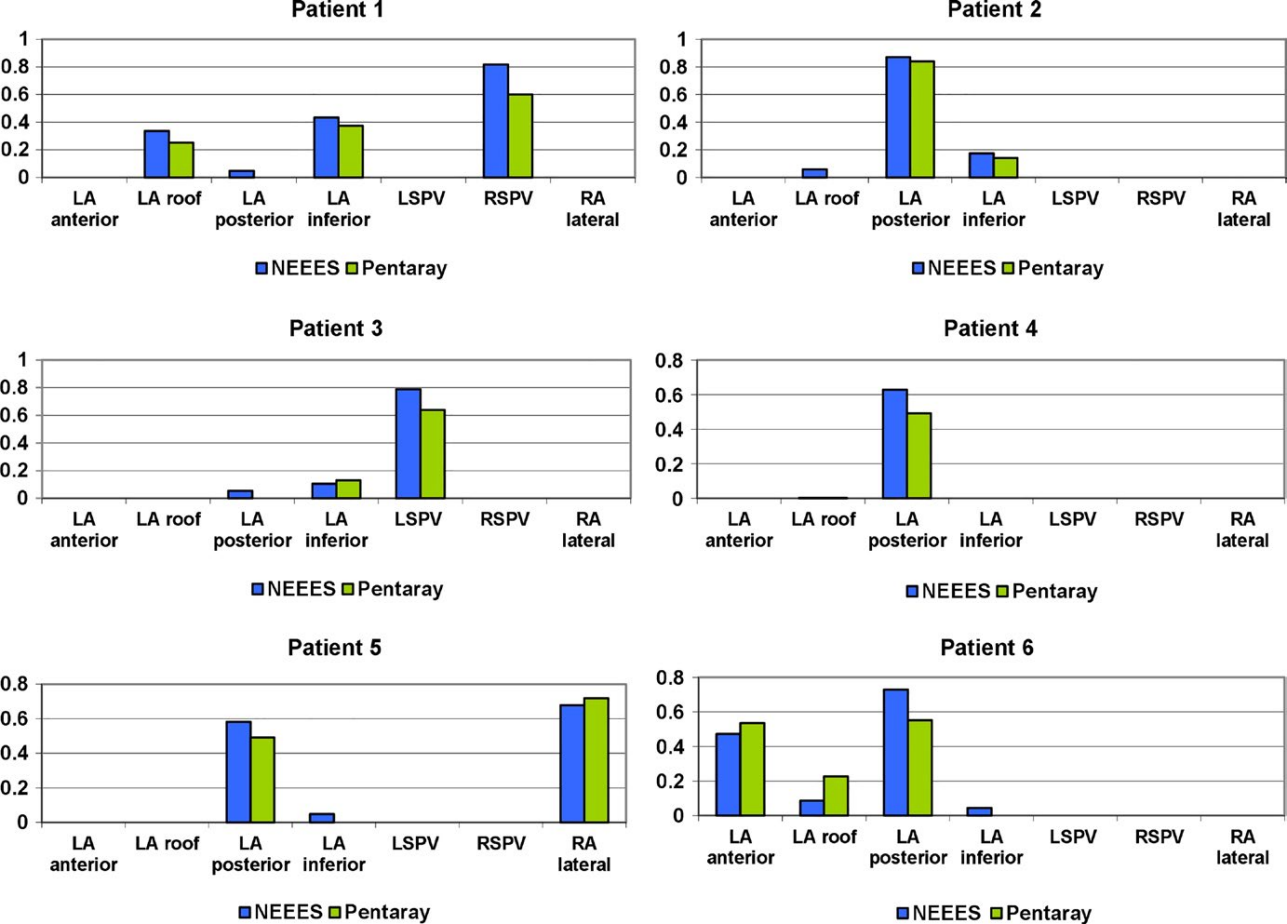
Rotor occurrence rates mapped noninvasively and invasively in atrial segments investigated by both mapping methods

**Figure 5:**
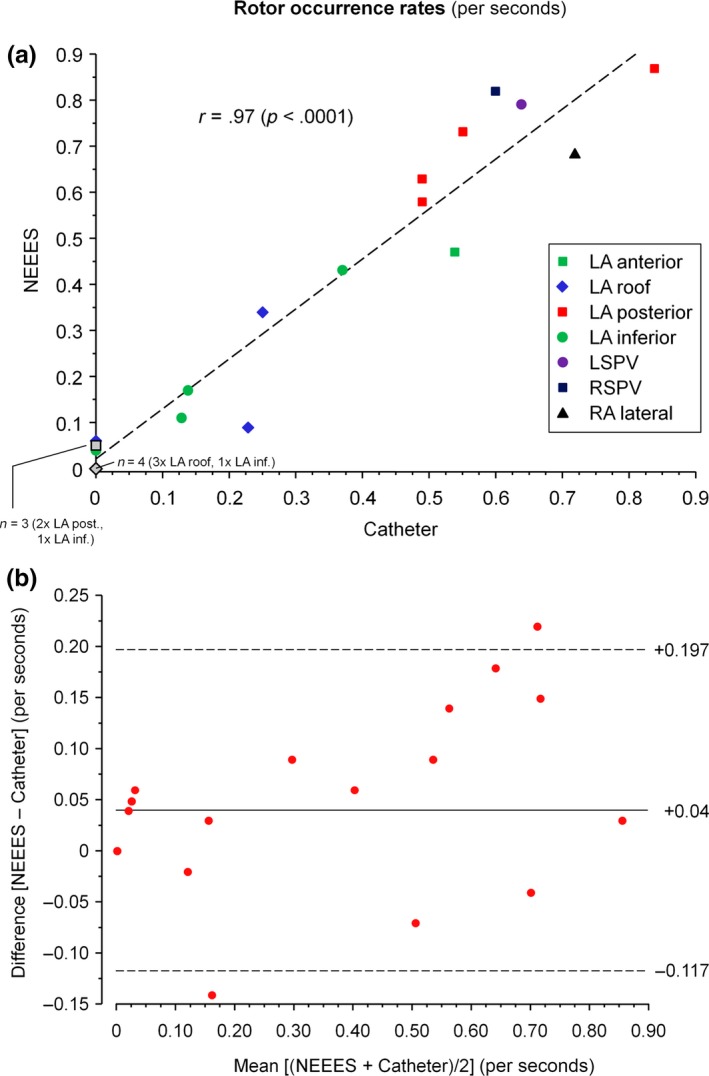
Statistical evaluation of rotor occurrence rate measurement. (a) Correlation of rotor occurrence rates as measured by the two techniques. LA, left atrium; LSPV, left superior pulmonary vein; RA, right atrium; RSPV, right superior pulmonary vein. (b) Bland–Altman plot of agreement between measurements of rotor occurrence rate by the two techniques. Mean difference (novel noninvasive epicardial and endocardial electrophysiology system [NEEES] – catheter) is 0.04 per second, with the zone of agreement ranging from −0.117 to 0.197 per second

Rotor occurrence rates obtained by NEEES and PentaRay mapping in atrial segments investigated by both types of mapping are given in Table 3 and presented graphically in Figure 4. Paired measurements were obtained from a total of 22 atrial segments. The correlation of measurements and the agreement between measurements are shown in Figures 5a and b, respectively. Spearman's correlation coefficient was 0.97, and the mean difference between the two techniques in rotor occurrence rates was 0.04 ± 0.08 per second, with lower and upper agreement limits of −0.117 and 0.197 per second.

The rotors which were detected at rotors aggregation sites were analyzed with special attention. Eighty‐seven rotors were detected noninvasively (during 123,182 msec of the total observation time) and 32 rotors detected by PentaRay mapping (during 49,122 msec of the total mapping period) at the dominant aggregation sites in the six patients. Therefore, the rotor occurrence rate was 0.65 per second by PentaRay mapping and 0.71 per second by noninvasive mapping at the dominant rotor aggregation sites. The properties of rotors observed with both mapping techniques at dominant rotor aggregation sites are presented in Table 4.

**Table 4:** Properties of rotors in dominant sites of rotor observation

	Patient 1		Patient 2		Patient 3		Patient 4		Patient 5		Patient 6	
Dominant site of rotor observation	RSPV		LA posterior		LSPV		LA posterior		RA lateral		LA anterior	
Mapping device	NEEES	Catheter	NEEES	Catheter	NEEES	Catheter	NEEES	Catheter	NEEES	Catheter	NEEES	Catheter
Observed rotors, *n*	17	5	15	6	15	5	15	5	14	6	11	5
CW rotors, *n* (%)	7 (41)	2 (40)	7 (47)	2 (33)	9 (60)	3 (60)	8 (53)	3 (60)	8 (57)	3 (50)	6 (55)	3 (60)
*p*‐value^a^,^b^	1.00		.66		1.00		1.00		1.00		1.00	
Full rotations, *n*	1.3 ± 0.4	1.4 ± 0.4	1.2 ± 0.4	1.2 ± 0.4	1.3 ± 0.4	1.4 ± 0.3	1.3 ± 0.4	1.2 ± 0.4	1.2 ± 0.4	1.3 ± 0.4	1.6 ± 0.7	1.7 ± 0.4
*p*‐value^a^,^c^	.61		1.00		.44		.57		.83		.59	
Cycle length, msec	144 ± 19	149 ± 17	168 ± 11	172 ± 9	192 ± 21	188 ± 21	166 ± 10	165 ± 8	157 ± 12	163 ± 9	156 ± 8	158 ± 6
*p*‐value^a^,^d^	.76		.31		.60		1.00		.36		.50	

LA, left atrium; LSPV, left superior pulmonary vein; NEEES, noninvasive epicardial and endocardial electrophysiology system; RA, right atrium; RSPV, right superior pulmonary vein.

a NEEES vs catheter.

b Fisher's exact test.

c Chi‐square test.

d Mann–Whitney *U* test.

The total number of rotors with clockwise rotation at these sites was 45 (52%) and 16 (50%), respectively. Per patient there were no statistically significant differences between the mapping techniques in the incidence of clockwise rotors and the numbers of full rotations; mean rotor cycle lengths were also not different.

## 4 DISCUSSION

Identification and localization of AF‐initiating triggers and the AF‐maintaining substrate is a prerequisite for effective AF treatment strategies either by surgical or catheter‐based ablation. Our study demonstrates that persistent AF patterns visualized by noninvasive phase mapping include frequent appearances of short‐lasting electrical rotors and comparatively few occurrences of focal‐type sources.

The rotors' spatial and temporal characteristics seem to be stochastic; however, the characterization of 30 and more rotor forming events in each patient enabled to identify some spatial regularity. Most of the rotors were observed at only a few atrial sites: 1 patient had 3 and the other five patients had two rotor aggregation sites.

The spatial distribution of the rotors demonstrated some common trends. In particular, in all patients, roughly two‐thirds of identified rotors were observed in the left atrium. Moreover, rotors were often observed in the posterior region of the LA, in areas near the superior PV and in the lateral wall of the RA. In contrast, rotors were rarely observed in the RAA and LAA, in areas near the inferior PV, the IVC ostium and on the atrial septum. The reasons for the heterogeneous spatial distribution of the rotors require further studies.

Nevertheless, the overall spatial distribution of atrial rotors appears to be patient‐specific.

Invasive phase mapping using a multi‐electrode catheter and applying the same mathematical algorithm confirmed the findings of noninvasive mapping. A close correlation was found for rotor aggregation sites as determined by either mapping technique. Rotor properties, such as rotor cycle length and numbers of full rotations also demonstrated pronounced similarities.

AF patterns observed in the current study do not contradict those observed in optical mapping studies (Hansen et al., 2015; Mandapati et al., 2000) and show close similarity to previously published data of noninvasive phase mapping of human AF (Haissaguerre et al., 2013, 2014). At the same time, rotors in our study differed from the rather stable rotors reported by Narayan et al. (2013). Both noninvasively and invasively detected rotors were rather short‐lasting. Characteristics such as average rotor cycle length and predominant rotors localizations within the LA were similar.

The differences in stability of rotors in this study, when compared to published data (Narayan et al., 2013), can be explained by two reasons. First of all, only short TQ ECG intervals were analyzed in order to avoid the influence of ventricular electrical activity. That could result in an underestimation of the rotor's lifetime. Moreover, different mathematical algorithms of signal processing were applied which might also affect the calculation of rotor lifetime.

In the current study, not only rotors but also focal sources with centrifugal activation were observed, similar to previous studies, in which identified focal drivers were used as ablation targets (Haissaguerre et al., 2013, 2014; Narayan et al., 2013). Focal‐type patterns during AF can be interpreted in various ways; either by real ectopic activity or by scroll waves as shown based on the 3D model of AF (Nash et al., 2006).

Noninvasive phase mapping has several methodological limitations. Phase mapping was initially developed for processing of action potential signals provided by optical mapping and mathematical simulations. Numerous studies provided evidence that the phase mapping based on action potentials can correctly image excitation patterns in reentrant cardiac arrhythmias (Gray et al., 1998; Hansen et al., 2015; Mandapati et al., 2000). Due to the fact that optical mapping is not available in humans, phase mapping was adopted for unipolar EGs assessed in noninvasive or invasive mapping studies. Previous studies have shown promising results for phase mapping based on unipolar EGs (Clayton & Nash, 2015; Narayan et al., 2013; Nash et al., 2006; Umapathy et al., 2010), but the similarity of the results provided by optical and electrical phase mapping has yet to be proved. Unlike phase mapping based on action potentials, phase mapping based on unipolar EGs require more stringent signal pre‐processing including band‐pass filtering which may decrease the spatial and temporal resolution of the method. This fact calls for comprehensive validation of phase mapping.

In this study, we showed that activation maps could not be constructed by contact bipolar EGs because of fractionation. Thus, we were not able to compare real cardiac excitation patterns with those provided by phase mapping. In this study, we only demonstrated a good correlation—in terms of spatial and temporal distribution of rotors and of their intrinsic properties—between invasive and noninvasive phase mapping in patients with atrial fibrillation. A deeper study of the issue calls for advanced experimental methods such as simultaneous electrical and optical mapping of Langendorff‐perfused hearts.

Noninvasive imaging has limited spatial resolution. According to our data, the accuracy in determining pacing origins at various locations around the atria is 7.4 ± 2.7 mm for the RA and 6.9 ± 2.3 mm for the LA (Wissner et al., 2017). In this regard, we cannot expect better precision in the localization of rotor cores. At the same time, the observed dynamics of the AF rotors can be considered a stochastic process. Identification of rotor aggregation sites using our methodology provides for a kind of averaging of rotor core locations over a long‐time period. This factor can compensate for the imprecision of noninvasive imaging.

In conclusion, the results of the current study demonstrate the equivalence of invasive and noninvasive phase mapping of rotors and focal activity when applying the same mathematical algorithm. However, the adequacy and reproducibility of the observed rotor and driver characteristics require further investigation.

### 4.1 Limitations

The multipolar catheter used in this study covers a maximum area of 9.6 cm^2^. Therefore, panoramic invasive mapping of the complete atria with a single application was not possible. Due to this limitation, the time synchronism between invasive and noninvasive phase mapping results could not be demonstrated. Our analysis is based on only 6 patients. Further investigations are necessary before final conclusions can be drawn.

## 5 CONCLUSIONS

In patients with persistent AF, phase processing of unipolar EGs recorded invasively by a 20‐pole mapping catheter could reproduce electrical rotors and focal activity as characterized by noninvasive phase mapping. The properties of the rotors detected by either technique demonstrated statistically significant spatial and temporal similarity. The key question still to be answered is whether the identification of rotors can serve as the basis for an ablation approach.

## CONFLICT OF INTEREST

E. Wissner and K.‐H. Kuck serve on the scientific advisory board of EP Solution SA. V. Kalinin owns stock in EP Solutions SA. A. Tsyganov is a consultant to EP Solutions SA.
